# Comparison of the influence of ticagrelor and clopidogrel on inflammatory biomarkers and vascular endothelial function for patients with ST-segment elevation myocardial infarction receiving emergency percutaneous coronary intervention: study protocol for a randomized controlled trial

**DOI:** 10.1186/s13063-016-1168-9

**Published:** 2016-02-11

**Authors:** Zhenhua Li, Yueyan Li, Tao Zhang, Wei Miao, Guohai Su

**Affiliations:** Department of Cardiology, Jinan Central Hospital, Shandong, China; Department of Orthopedics, Jinan Central Hospital, Shandong, China

**Keywords:** Ticagrelor, Clopidogrel, ST-segment elevation myocardial infarction (STEMI), Anti-inflammatory, Circulating endothelial cells (CECs)

## Abstract

**Background:**

The Platelet Inhibition and Patient Outcomes (PLATO, Eur J Prev Cardiol 22(6):734–42, 2015) trial shows that, in patients who have an acute coronary syndrome, treatment with ticagrelor as compared with clopidogrel significantly reduced the rate of death, but the reason is still uncertain. Both inflammation and vascular endothelian cell dysfunction play important roles in the pathophysiology of atherosclerotic plaques, but whether ticagrelor has superior anti-inflammatory effect and can improve vascular endothelial cell function to a great extent is unknown.

**Methods/design:**

Patients with STEMI who are scheduled to undergo emergency percutaneous coronary intervention (PCI) will be randomly assigned to receive a loading dose of ticagrelor 180 mg as the treatment group or clopidogrel 600 mg as the control group. After PCI, the treatment group will be treated with ticagrelor 90 mg twice daily while the control group will be treated with clopidogrel 75 mg once daily. The vascular endothelial function will be tested by circulating endothelial cells, and levels of inflammation will be tested by CD40 ligand (CD40L), high sensitivity C-reactive protein (hsCRP) and P-selectin. The estimated enrollment sample size will be 350 patients, including 175 in the treatment group and 175 in the control group.

**Discussion:**

This study will compare the influence of ticagrelor and clopidogrel on inflammatory biomarkers and vascular endothelial function firstly for STEMI patients receiving emergency PCI and will provide evidence to identify whether ticagrelor inhibits inflammation and improves vascular endothelial cell function to a greater extent than clopidogrel or not.

**Trial registration:**

This trial was registered with Clinicaltrials.gov (identifier: NCT02123004) on 20 April 2014.

## Background

Acute myocardial infarction (AMI) is a serious illness hazard to human health and has a high mortality rate. It has been shown that atherosclerotic plaque rupture causes serious clinical events. In recent years, the criminal vascular patency rate has been significantly improved by applying emergency PCI treatment, but it is still lacking in restoration and protection of damaged myocardium. Inflammation is a key factor in the development of atherosclerotic disease and acute coronary syndrome (ACS) [2–5]. Currently, it is considered that plaque rupture caused by the joint action of inflammation and vascular endothelial cell dysfunction leads to AMI, and the concentration of inflammatory markers is closely related to the degree of instability and progression of atherosclerotic plaque [6]. In the review published in *Nature Medicine*, hypercholesterolemia and inflammation were described as “partners in crime” [7]. Therefore, under the premise of recanalization of occlusions and revascularization, regulating inflammatory reactions of lesions is the key to improving the prognosis of patients with AMI.

Platelets participate in the process of forming and extending atherosclerotic plaques, and are a source of inflammatory mediators [8]. The activation of platelets by inflammatory triggers may be a critical component of atherothrombosis [9]. Now more and more evidence has proven that the processes of inflammation and thrombosis are closely related [10, 11]. Since platelet activation, thrombosis and inflammation are closely linked, effective platelet inhibition may also bring about an anti-inflammatory effect. Therefore, anti-platelet medicines should have other functions in addition to anticoagulant effects, and it has been shown that they have direct or indirect anti-inflammatory effects in vitro or in vivo [12–14].

Studies have shown that aspirin can inhibit transforming growth factor-β and proliferation of vascular smooth muscle cell [15, 16], and it is able to lower the activity of nuclear factor-κB, which is necessary in coding proinflammatory mediators such as monocyte chemoattractant protein-1 and interleukin-8 [17, 18].

Independent of the loading or maintenance dose, patients treated with prasugrel, one of the antiplatelet medicines, exhibited significantly more potent platelet inhibition as determined by adenosine diphosphate, and collagen-induced aggregation, Ultegra Analyser measurements, and surface expression of platelet endothelial cell adhesion molecule-1, platelet glycoproteinIIb/IIIa antigen, and activity with procaspase activating compound-1 antibody, glycoprotein Ib, P-selectin, CD40L, platelet glycoprotein 37, and thrombospondin receptor expression when compared with those treated with clopidogrel [19]. Prasugrel use with a 60 mg loading dose and 10 mg maintenance dose inhibits several markers of platelet activation and the formation of platelet-monocyte aggregates more effectively than a 600 mg loading dose and 75 mg maintenance dose of clopidogrel [20].

Another antiplatelet medicine, clopidogrel, has been proved to lower the activities of, for example, inflammatory cytokines, C-reactive protein (CRP), and P-selectin [12, 13, 21–24]. There is also substantial evidence that therapy with clopidogrel can lead to reductions in serum levels of CD40L, CRP, P-selectin, and platelet-leukocyte aggregate formation in patients with ischemic vascular disease, especially in patients with ACS receiving PCI [25]. Chen et al. compared aspirin alone with aspirin plus clopidogrel (loading dose of clopidogrel 300 mg, followed by clopidogrel 75 mg daily) for effects on high sensitivity C-reactive protein (hsCRP) in 115 patients with non-ST-segment elevation acute coronary syndrome (NSTE-ACS) [26]. Mean CRP levels were reduced at both 7 and 30 days in both groups, and clopidogrel was significantly more effective than aspirin alone in lowering CRP.

Integrity loss of vascular endothelial cells is considered to be a consistent factor of atherosclerotic plaque rupture and non-atherosclerotic vascular wall rupture events [27–29]. The number of circulating endothelial cells (CECs) in many vascular injury diseases is significantly elevated [30, 31]. Oxidative stress-mediated endothelial cell injury and a concurrent increase in CECs are seen in many cardiovascular diseases, including myocardial infarction, unstable angina [32], and coronary artery disease [33]. Damani et al. [34] have characterized CECs using the first automated and clinically feasible CEC three-channel fluorescence microscopy assay in 50 consecutive patients with STEMI and 44 consecutive healthy controls. CEC counts were significantly elevated in myocardial infarction cases versus controls, with median numbers of 19 and 4 cells/ml, respectively. CECs from patients with STEMI are heterogeneous in size and shape and many of them are much larger than CECs from controls, with multiple nuclei.

Recent work has suggested that, in addition to its primary effect on platelet function, clopidogrel has anti-inflammatory capability [25] and enhances endothelial production of nitric oxide and prostacyclin in tissue culture [35]. This, in concert with its ability to inhibit CD40L both in vitro and in vivo [36], suggests that clopidogrel possesses the ability to preserve endothelial function by a mechanism that might be independent of its antiplatelet activity. Among other effects, CD40L blockade stimulates heme oxygenase 1 expression [37]. McClung et al. [38] observed the influence of clopidogrel treatment on endothelial function in diabetic patients. The patients with diabetes mellitus had an elevated number of CECs (mean 79 ± 15 cells/ml of peripheral blood, range 20 to 110). After 4 weeks of therapy with clopidogrel, 75 mg/day, a marked reduction in the number of CECs was observed in all subjects to within the normal range (mean 10 ± 4 cells /ml, range 0 to 40; *p* < 0.001).

Ticagrelor is an oral, reversible, direct-acting inhibitor of the adenosine diphosphate receptor P2Y12 that has a more rapid onset and more pronounced platelet inhibition than clopidogrel. The Platelet Inhibition and Patient Outcomes (PLATO) trial shows that, in patients who have an ACS, treatment with ticagrelor as compared with clopidogrel significantly reduced the rate of death from vascular causes, myocardial infarction, or stroke. Although several studies have attempted to illustrate the reason for the results of the PLATO trial, such as stating that ticagrelor is dependent on adenosine receptor activation [39], as of yet there is no identified evidence. So what are the roles of an anti-inflammatory and the CEC enhancing capability? Is ticagrelor superior to clopidogrel in these two ways?

The DISPERSE 2 (Dose Confirmation Study Assessing Anti-Platelet Effects of AZD6140 vs Clopidogrel in NSTEMI 2) trial [40] has compared ticagrelor with clopidogrel in anti-inflammatory ability. This trial included patients who were hospitalized for NSTE-ACS within the 48 h before study entry, had ischemic symptoms ≥10 min at rest, and had either biochemical marker evidence of myocardial infarction or electrocardiographic evidence of ischemia. The objective of the analysis from the DISPERSE 2 trial was to compare ticagrelor and clopidogrel for effects on the inflammatory biomarkers CRP, interleukin 6, myeloperoxidase, and soluble CD40 ligand. The results showed that CRP levels had increased somewhat at discharge and were reduced only at 4 weeks. Ticagrelor and clopidogrel appeared not to differ in this study with respect to the inflammatory biomarkers in patients with NSTE-ACS [41]. Differences in timing of the measurements, treatment regimens, or patient conditions could have affected the results.

As we know, STEMI has a very different pathology compared with NSTE-ACS [42, 43]. Although numerous relevant experiments have shown that antiplatelet drugs have direct or indirect anti-inflammatory and endothelial protective effects, whether ticagrelor is superior to clopidogrel in anti-inflammatory effect and protection of vascular endothelium is not clear. To the best of our knowledge, this study will be the first one to compare the influence of these two drugs on inflammatory biomarkers and vascular endothelial function for patients with STEMI receiving emergency PCI.

## Methods

### Study design

This study (https://clinicaltrials.gov/ct2/home registration: NCT02123004) is a prospective, open-label, multicenter, clinical study using continuous monitoring with a clinical research organization.

The study will be conducted at three clinical centers in China. Up to 350 patients will be enrolled. Those enrolled will be assumed to be patients with STEMI who are scheduled to undergo emergency PCI. The inclusion and exclusion criteria are showed in Tables 1 and 2, respectively.

**Table 1:** Inclusion criteria

For inclusion in the study the subject should fulfill the following criteria:
a) Male or non-pregnant female
b) Age ≥ 18 years old and <80 years old
c) Consecutive patients who should be hospitalized with documented evidence of STEMI receiving PCI
d) All patients have persistent ⩾ 0.2 mV ST-segment elevation in two or more contiguous precordial leads or ⩾ 0.1 mV ST elevation in two or more contiguous limb leads, with one of the following: persistent chest pain or elevation of biomarkers of myocardial necrosis
e) Time from chest pain onset to receiving PCI <12 h
f) Persistent chest pain <12 h
g) Provision of informed consent prior to any study-specific procedures.

**Table 2:** Exclusion criteria

Subjects should not enter the study if any of the following exclusion criteria are fulfilled:
a) Involved in other trials
b) In last year have P2Y12 receptor antagonist drug treatment history or long-term use of immunosuppressive agents
c) Recurrent myocardial infarction or previous history of CABG surgery or rescue PCI
d) Active bleeding or bleeding history
e) Obvious infection and body temperature (axillary temperature) higher than 38.0 °C
f) Autoimmune diseases
g) Malignancies
h) In last 6 months have received major surgery
i) Left ventricular ejection fraction is less than 30 %
j) Life expectancy less than one year.

Enrolled patients will be followed up for ≥ 4 weeks, with four visits during hospitalization and one visit after discharge. During follow-up, blood samples will be collected.

### Ethical conduct of the study and informed consent

Ethical approval was obtained from the Ethical Review Board of Jinan Central Hospital, the Ethical Review Board of the People’s Hospital of Shandong Provence, and the Ethical Review Board of the People’s Hospital of Dezhou City. The trial will be performed in accordance with ethical principles according to the Declaration of Helsinki and are consistent with Good Clinical Practice a applicable regulatory requirements. They will also be conducted in compliance with applicable local laws and regulations of each participating center, including data protection laws, and any requirements imposed by local competent authorities, ethics committees, and institutional review boards.

Informed consent is obtained from every participant in the trial.

### Objectives

The trial objectives are a) to observe whether ticagrelor inhibits inflammation and improves vascular endothelial cell function to a greater extent than clopidogrel in STEMI patients whether they receive PCI or not, and b) to observe whether ticagrelor can reduce the serum levels of inflammatory biomarkers both in coronary and in peripheral venous in patients with STEMI or not.

### Safety objective

The safety objective of this study in patients will be evaluated by the occurrence of any adverse event (AE) during the 4 weeks follow-up: Suspected bleeding/reinfarction/rehospitalization/revascularization by PCI or coronary artery bypass graft/sudden death/stroke/allergic or allergic-like reactions, or other AEs and serious adverse events (SAEs).

### Study procedures

**Fig. 1:**
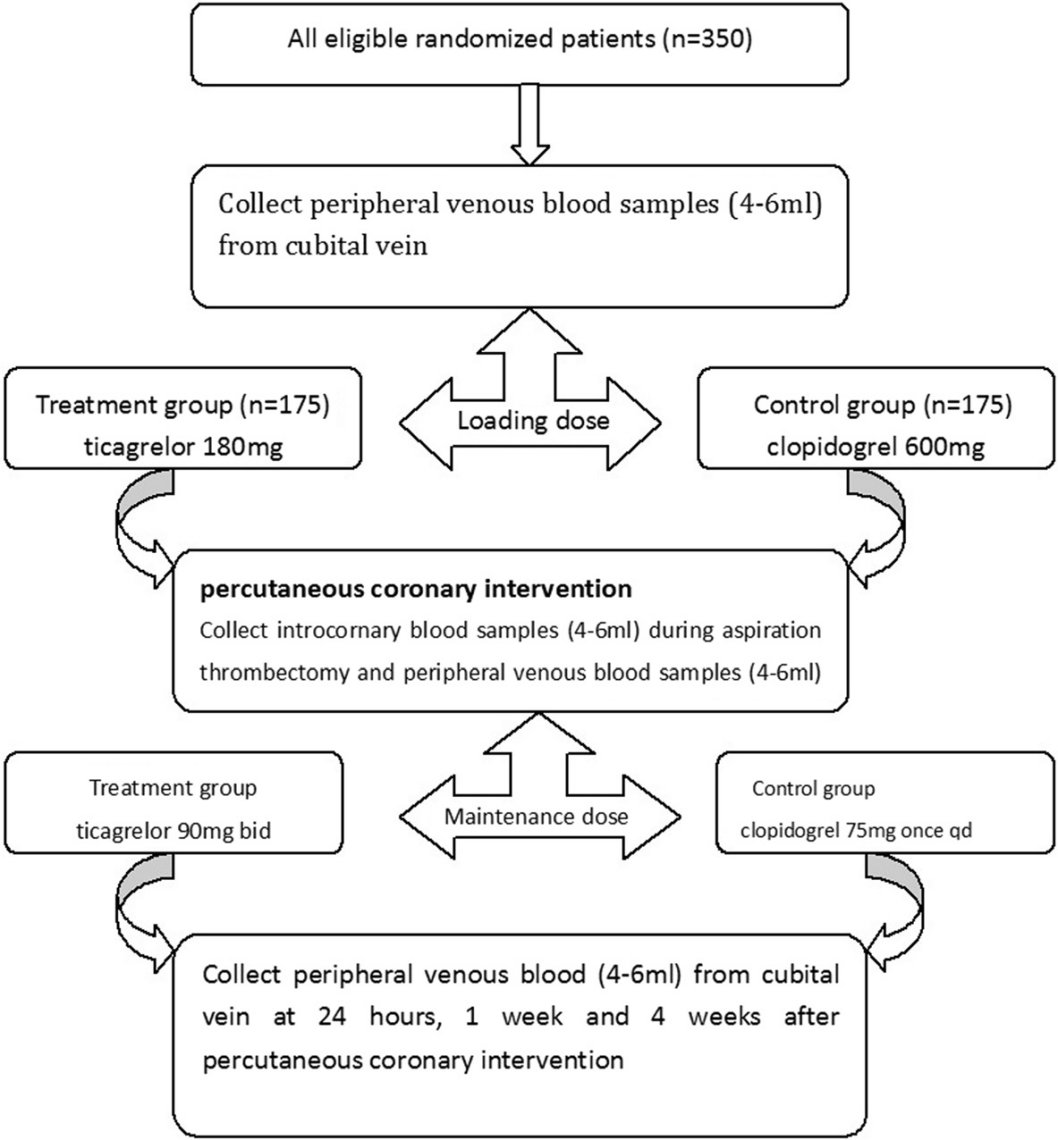
Study procedure

Patients who are scheduled to undergo emergency PCI will be randomly assigned to receive a loading dose of ticagrelor 180 mg as the treatment group or clopidogrel 600 mg as the control group. After PCI, the treatment group will be treated with ticagrelor 90 mg twice daily while the control group will be treated with clopidogrel 75 mg once daily. The vascular endothelial function will be tested by CECs, and levels of inflammation will be tested by CD40L, hsCRP and P-selectin (see Fig. 1).

### Collection of study variables

#### Recording of data

At visit 1, subjects who provide informed consent for the study will be assigned a number. After patients meet all study entry criteria and report to the research unit for the first treatment period, a unique three-digit subject number from the randomization code is assigned to each subject.

#### Data collection at enrollment and follow-up

Each patient will undergo enrollment procedures at visit 1, and all the variables recorded in the clinical chart will be source data when the patient is in the hospital. At the day of discharge, the patients will receive a patient diary to record investigational medication information and will be told the follow-up visit date for the laboratory test and clinical examination. Patients will have routine visits as outlined in Table 3.

**Table 3:** Data to be collected at routine visits

	Screening phase	Study phase				
Evaluation	Screening	Follow-up				
Time	−30 min	0	1 hour (after PCI)	24 hours (after PCI)	1 week (after PCI)	4 weeks (±3 days) (after PCI)
Visit number	V0	V1	V2	V3	V4	V5
Design						
Inclusion/exclusion criteria	x					
Informed consent	x					
Demographics	x					
Medical/surgical history, allergy history, alcohol habits, smoking habits	x					
Symptom inquiry	x	x	x	x	x	x
Body weight	x					x
Physical examination	x	x	x	x	x	x
Vital signs: temperature/pulse/respiration/blood pressure	x	x	x	x	x	x
18-lead ECG	x	x	x	x	x	x
AE/SAE recording	x	x	x	x	x	x
Treatment adherence	x	x	x	x	x	x
Myocardial necrosis biomarkers	x			x	x	x
Laboratory testing/efficacy						
CD40l/CRP/P-selectin/CECs		x		x	x	x
Laboratory testing/safety						
Creatinine		x		x	x	x
Uric acid				x	x	x
AST/ALT/TBIL				x	x	x
Blood-routine		x		x	x	x
TG/TC/HDL-C/LDL-C				x	x	x
Serum Na^+^, K^+^, Cl^−^		x		x	x	x
Urinary albumin				x		x

Evaluations that are reported in the Case Report Form must be supported by appropriately signed identified source documentation related but not limited to the following:

- Agreement, date, and signature of informed consent mentioning the study identification.
- Patient identification, last participation in a clinical trial, medical history, associated diseases, and data related to the studied pathology.
- Previous and concomitant medication.
- Study identification.
- Treatment number, dates of administration.
- Dates of visits and assessments including the examination report.
- Vital signs, height, body weight. ECG records signed and dated.
- Adverse events and follow-up:

In the case of an SAE, the site should file in the source document at least copies of the hospitalization reports and any relevant examination reports documenting the follow-up of the SAE.

- Date of premature study discontinuation (if any) and reason.

Source documentation may be found in the following:

- Patient’s identity.
- Medical history.
- Hospital records.

### Statistical methods and sample size determination

#### Description of analysis sets

The primary analysis was proposed to be based on the intention-to-treat principle. All randomized patients who had at least one dose of investigational medication would be included. If a patient withdrew their informed consent after randomization without taking any medication, he/she would be excluded from the analysis. The analysis set according to the above principle was defined as a modified full analysis set (mFAS).

If there were other major protocol violations, after the adjudication by the principle investigator, the corresponding patients would be further excluded from the per protocol analysis set (PPS).

#### Efficacy analysis set

The efficacy analysis will be conducted on both the mFAS and PPS.

#### Safety analysis set

The safety analysis would be conducted on the mFAS.

### Methods of statistical analyses

Categorical variables were described by counts and proportions, and continuous variables were described by median and interquartile range. Proportions were compared by chi-square tests and continuous outcome by Mann-Whitney U tests. A two-sided level of significance of 0.05 was applied to general comparison.

To avoid the multiplicity issue induced by multiple endpoints, the Bonferroni-Holm’s method will be used to control the overall alpha level. All analyses will be performed using SAS 9.1.3 (SAS Institute Inc., Cary, NC, USA).

### Determination of sample size

- The key endpoints of this study were the changes of hsCRP, CD40L, P-selectin, and CECs from baseline to 4 weeks after PCI. The relevant data was limited in the published literature. For patients with NSTE-ACS [41] the change of inflammatory markers at 4 weeks had not been observed and the variation of markers was huge.
- In our study, the target population was patients with STEMI and PCI. We assume the inflammatory and endothelial function markers would be different for ticagrelor and clopidogrel after 4 weeks of treatment. This study was proposed to detect a moderate effect size, which means the expected standardized difference (mean difference/common standard deviation) between the two treatment groups was 0.4.
- The fact that there were four key endpoints in this study led to the multiplicity issue. To control the overall type I error, the significant level for each endpoint was set to 0.0125 (0.05/4). Under the above assumptions, with a two-sided 0.0125 alpha level and a 20 % dropout rate, 350 patients (175 in each group) in total would offer 80 % power to detect the estimated moderate difference between ticagrelor and clopidogrel among any of the primary endpoints.

### Audits and inspections

Authorized representatives of the institution, a regulatory authority, or an ethics committee may perform audits or inspections at the center, including source data verification.

## Discussion

For reasons of economy and the medical care system, clopidogrel is still the most widely used antiplatelet medicine in ACS in addition to aspirin [44]. A number of individuals experience clopidogrel resistance, which leads to adverse thrombotic events [45]. Although a study shows that the most common cause of clopidogrel’s failure in treatment is medication nonadherence, inter-individual variability in response to clopidogrel is also an important reason, which is appreciated in up to 30–40 % of patients who received clopidogrel treatment [46–48]. As another P2Y12R antagonist, ticagrelor is becoming a strong competitor to clopidogrel.

Although much progress has been made in the treatment of STEMI, there is still high mortality of patients in the real world. As a phase III clinical trial, the PLATO trial compared ticagrelor to clopidogrel in addition to aspirin in over 18,000 patients with ACS. Results show a significant decrease in primary efficacy outcome (composite endpoint of death from vascular causes, myocardial infarction, or stroke) at 12 months (9.8 % with ticagrelor versus clopidogrel 11.7 %; *p* < 0.001) [49]. The incidence of stent thrombosis across definitions was reduced in patients receiving ticagrelor compared with clopidogrel regardless of type of ACS, presence of diabetes, and stent type [50].

The pathophysiology of ACS is multifactorial. It involves disruption of vulnerable plaque and thrombus formation, which produces severe myocardial ischemia and downstream embolization in the coronary vascular bed, leading to subendocardial or transmural necrosis. All the above occur in response to inflammation [51, 52]. STEMI is caused by acute total coronary occlusion, whereas NSTE-ACS is associated with vulnerable plaque and subocclusive thrombosis. Although the  DISPERSE trial did not find the difference between the two drugs in NSTE-ACS patients, the big pathology gap between STEMI and NSTE-ACS deserves more attention to find the difference in the effects of these two drugs on inflammation and endothelial dysfunction in STEMI patients [41]. In addition, more STEMI patients received emergency PCI compared with NSTE-ACS patients, and STEMI patients will also experience ischemia followed by rapid reperfusion and are consequently at risk of ischemia/reperfusion injury, which will lead to high levels of inflammation [53]. After PCI, patients always show better compliance in taking antiplatelet medicine than no-PCI patients, so the antiplatelet medicine’s long effect in treatment will be observed in this trial.

To the best of our knowledge, this study is the first study to compare the influence of ticagrelor and clopidogrel on inflammatory biomarkers and vascular endothelial function for patients receiving emergency PCI and will provide evidence to identify whether or not ticagrelor inhibits inflammation and improves vascular endothelial cell function to a greater extent than clopidogrel.

## Trial status

Recruitment began in May 2014 and is ongoing. Seventy patients have been recruited.

## Abbreviations

ACS: acute coronary syndrome
AE: adverse event
AMI: acute myocardial infarction
CD40L: CD40 ligand
CEC: circulating endothelial cell
CRP: C-reactive protein
mFAS: modified full analysis set
NSTE-ACS: non-ST-segment elevation acute coronary syndrome
PCI: percutaneous coronary intervention
PLATO: Platelet Inhibition and Patient Outcomes trial
PPS: per protocol analysis set
SAE: serious adverse event
STEMI: ST-segment elevation myocardial infarction
